# A case for eliminating Widal testing for enteric fever, India

**DOI:** 10.2471/BLT.26.295696

**Published:** 2026-10-01

**Authors:** Kamini Walia, Ram Gopalakrishnan, Priscilla Rupali, Camilla Rodrigues, V Ramasubramanian, Veeraraghavan Balaji, Vinod Ohri, Navin Dang, Chand Wattal, Himanshu Reddy, Nitin Bansal, Hina Singh

**Affiliations:** a Division of Descriptive Studies, Indian Council of Medical Research, Ansarinagar, New Delhi - 110029, India.; b Department of Infectious Diseases, Apollo Hospitals, Chennai, India.; c Department of Clinical Microbiology, Christian Medical College and Hospital, Vellore, India.; d Department of Microbiology, PD Hinduja National Hospital, Mumbai, India.; e Dang Pathology Laboratory, New Delhi, India.; f Institute of Clinical Microbiology and Immunology, Sir Ganga Ram Hospital, New Delhi, India.; g Department of Medicine, King George's Medical University, Lucknow, India.; h Department of Infectious Diseases, Rajiv Gandhi Cancer Institute and Research Centre, New Delhi, India.

Enteric fever remains a preventable yet persistent public health challenge in many low- and middle-income countries, particularly in the African and South-East Asia regions.^1^ The disease caused by *Salmonella enterica* serovars Typhi and Paratyphi disproportionately affects children, adolescents and young adults, with an estimated global burden of over 14 million cases and nearly 136 000 deaths annually.^1^ India carries a substantial proportion of this burden. Hospital-based surveillance in India reports incidence ranging from 12 to 1622 cases per 100 000 child-years among children and 108 to 970 cases per 100 000 person-years among people older than 15 years, highlighting both high burden and substantial uncertainty in measurement.^2^ Despite advances in treatment, vaccination and surveillance, progress towards accurate, accessible diagnostics for enteric fever remains limited.^3^

Accurate diagnosis is essential for controlling infectious disease, yet the link between poor diagnostics and antimicrobial resistance remains underappreciated. False-positives lead to unnecessary antibiotic exposure, while false-negatives delay appropriate treatment and encourage the use of broader-spectrum antibiotics without microbiological confirmation. Both scenarios drive drug resistance. This problem is especially acute for enteric fever in endemic settings, where its clinical presentation overlaps with malaria, dengue, scrub typhus, chikungunya, leptospirosis and other viral fevers. Poor diagnostics affect patient care and weaken surveillance by hindering accurate assessment of disease burden and transmission patterns, compromising vaccine policy and investment decisions. Reliable diagnostics are therefore essential for both patient care and evidence-based public health policy-making. Here, we argue that eliminating inaccurate serological tests, particularly the Widal test, is a needed and achievable intervention for containing the emerging resistance.

In India, where *S*. *Typhi* already shows widespread antibiotic resistance,^2^ continued reliance on inaccurate diagnostics is harmful. Of particular concern is the near-global dissemination of multidrug-resistant lineages of *S. Typhi*, with inappropriate prescribing driven by inaccurate diagnostics further accelerating the emergence and spread of extensively drug-resistant strains.^4^ Blood culture remains the benchmark for diagnosis of enteric fever and provides the added benefit of antimicrobial susceptibility data. However, the sensitivity of blood culture is moderate (50–65%) and is affected by prior antibiotic use,^5^ the timing of sample collection and blood volume. Uneven distribution of laboratory infrastructure, availability of trained personnel, and quality assurance systems at primary care and district levels further constrain the effectiveness of blood culture.

The Widal test is a widely used diagnostic tool for enteric fever. The test detects antibodies against *S. Typhi* O and H antigens; however, it lacks validated cut-offs for acute disease and shows highly variable sensitivity and specificity in endemic settings.^5^ The test’s performance is affected by pre-existing antibodies in the population, cross-reactivity with other infections and inability to distinguish acute infection from past exposure.^3^ The Widal test can be performed as a slide agglutination or a tube agglutination test. The slide test is widely used across India, but well-established thresholds for determining a positive result are lacking. As a result, any visible reaction may be interpreted as a positive result, leading to false-positive diagnoses and unnecessary antibiotic treatment. The tube test, more commonly used in tertiary care centres, can provide more useful information when two blood samples taken at different stages of illness are compared to detect a rise in antibody levels. However, when performed on a single blood sample, as is often the case in routine practice, the tube test has limited value for diagnosing acute typhoid.

The largest comparative evaluation of typhoid diagnostics to date strengthens support for discontinuing the Widal test.^5^ An analysis showed that Widal performs poorly relative to other diagnostics and adds no incremental value when combined with better-performing rapid tests; notably, none of the best-performing diagnostic combinations included Widal.^5^ These findings align with a systematic review which concluded that no currently available rapid diagnostic test can reliably replace blood culture.^3^ Collectively, the evidence suggests that Widal is both inaccurate and redundant, providing a strong rationale for its phased withdrawal and regulatory restriction. Eliminating Widal would reduce reliance on a poorly performing diagnostic, limit inappropriate antibiotic use resulting from false-positive results and encourage investment in more accurate and reliable typhoid diagnostics.

A retrospective audit of 100 febrile children at a New Delhi tertiary hospital found that, among 54 Widal-positive patients, only five (9.3%) were confirmed by blood culture, while 49 (90.7%) were culture-negative.^6^ These findings illustrate the potential consequences of widespread reliance on Widal testing in high-volume settings. In a district hospital outpatient department managing about 1000 febrile patients each month, even a modest false-positive rate could result in many patients being misdiagnosed with typhoid and receiving unnecessary antibiotics, while alternative causes of fever may be missed. Across India, such reliance on Widal testing could therefore contribute to misdiagnosis, inaccurate disease surveillance and avoidable antibiotic use.

Despite longstanding evidence of poor performance and guidance from the World Health Organization (WHO) emphasizing microbiological confirmation, and the United States Centers for Disease Control and Prevention recommending against Widal serology, Widal testing remains widespread in endemic settings.^7^ In Pakistan, the Khyber Pakhtunkhwa province has issued directives to discontinue Widal testing, although enforcement has been inconsistent. Overall, formal regulatory action to restrict Widal testing remains limited, and the test continues to be widely used across many endemic countries, as reliable and affordable diagnostic alternatives remain elusive. India’s National Essential Diagnostics List, developed by the Indian Council of Medical Research in alignment with WHO guidance, promotes diagnostics with proven accuracy and does not include the Widal test.^8^ However, exclusion from the list only limits public-sector procurement; Widal remains legally available through India’s Central Drugs Standard Control Organisation licensed channels. Widal is widely used in the private sector, reflecting limited clinician awareness and gaps in state-level procurement practices. Eliminating Widal testing will require additional regulatory action by the organization, alongside procurement reforms, clinician education and enforcement measures.

India has previously confronted and successfully addressed a similar challenge by banning serological tests for tuberculosis in 2012. More than 1.5 million tuberculosis serological tests, generating an estimated 15 million United States dollars (US$) market, were done each year before this ban, largely in the private sector. Following WHO’s 2011 recommendation against their use, India issued a legally binding notification under the Drugs and Cosmetics Act prohibiting the manufacture, sale, distribution and use of tuberculosis serology kits.^9^ However, the ban alone was insufficient. Success required active enforcement, monitoring against substitution with other inappropriate tests, and parallel roll-out of a WHO-recommended cartridge-based nucleic acid amplification test through India’s national tuberculosis elimination programme. Combined with mandatory notification and the Nikshay surveillance system, this roll-out transformed tuberculosis and rifampicin-resistance detection, improved case notification including in the private sector, reduced inappropriate treatment and advanced public–private integration.^10^ The tuberculosis experience shows that eliminating an inaccurate diagnostic requires coordinated normative guidance, regulatory prohibition, validated replacement technology and enforcement even in fragmented health systems. Applying similar principles to enteric fever could yield comparable gains in clinical care, antimicrobial resistance containment and surveillance quality, though unlike for tuberculosis, no rapid, accurate test yet exists to immediately replace Widal.

Given the paucity of alternative rapid diagnostic tests, strengthening blood culture capacity at all levels of care is indispensable and has been recommended by the Indian Council of Medical Research. However, such strengthening alone may not suffice, as most febrile patients are managed in peripheral settings with limited access to microbiological testing. What is needed is a diagnostic ecosystem combining culture-based confirmation with validated, affordable point-of-care tools.

Several novel diagnostic approaches are under development. Molecular assays, targeting *Salmonella Typhi-*specific genes, offer high analytical sensitivity but face technical challenges related to low bacterial count in blood, cost and infrastructure requirements.^11^ Diagnostics based on host response show promise in differentiating enteric fever from other febrile illness, aligning with syndrome-based diagnostic approaches.^11^ IgA-based serological assays against antigens such as hemolysin E have demonstrated improved specificity in endemic populations.^12^ Antigen-detection and metabolomic approaches are also being explored for rapid, culture-independent diagnosis suitable for primary care.^11^

However, new innovations must be rigorously validated. Many promising diagnostics fail to translate from research to practice due to inadequate evaluation in real-world settings, lack of affordability or poor integration into health systems. Premature deployment of inadequately validated tests generates unnecessary follow-up testing, inappropriate treatments, hospitalizations and referrals. These wasted resources are especially costly where health budgets are limited. The *Lancet* Commission on Diagnostics frames such diagnostic inefficiencies within a broader diagnostics gap that undermines efficient use of health resources and equity.^13^ Policy-makers should recognize the elimination of inaccurate diagnostics for enteric fever as a priority intervention within global antimicrobial resistance and universal health coverage agendas. Six key actions will be helpful in phasing out unreliable tests. First, clear normative guidance from WHO and national authorities explicitly discouraging the use of the Widal slide test and similar poorly performing tests. Second, clear guidance about when and how to perform the Widal tube test correctly, and wide dissemination of this information. Third, regulatory action to restrict manufacture, distribution and procurement of inaccurate diagnostics. Fourth, investment in laboratory systems, including blood-culture capacity and quality assurance. Fifth, support the development of affordable, high-performance tests tailored to endemic settings, alongside rigorous analytical and field evaluation using standardized, scientifically robust protocols. Finally, clinician and public engagement to shift diagnostic practices. 

The absence of an accurate and affordable point-of-care typhoid diagnostic that can reliably replace blood culture exemplifies the diagnostic neglect surrounding enteric fever. While vaccines, antibiotics and surveillance systems have advanced, diagnostics for diseases endemic in low- and middle-income countries have remained stagnant, often to the detriment of patients and health systems. Continued reliance on inaccurate tests such as the Widal test undermines efforts to control enteric fever and contain antimicrobial resistance. Aligning diagnostic practice with scientific evidence is essential for patient safety, antimicrobial stewardship and credible public health data. India’s experience highlights both the importance of the problem and the feasibility of solutions. Eliminating inaccurate diagnostics is not a technological challenge but a policy choice.

## References

[1] Stanaway JD, Reiner RC, Blacker BF, Goldberg EM, Khalil IA, Troeger CE, et al.; GBD 2017 Typhoid and Paratyphoid Collaborators. The global burden of typhoid and paratyphoid fevers: a systematic analysis for the Global Burden of Disease Study 2017. Lancet Infect Dis. 2019 Apr;19(4):369–81. 10.1016/S1473-3099(18)30685-6 30792131 PMC6437314

[2] John J, Bavdekar A, Rongsen-Chandola T, Dutta S, Gupta M, Kanungo S, et al.; NSSEFI Study Team. Burden of typhoid and paratyphoid fever in India. N Engl J Med. 2023 Apr 20;388(16):1491–500. 10.1056/NEJMoa2209449 37075141 PMC10116367

[3] Wijedoru L, Mallett S, Parry CM. Rapid diagnostic tests for typhoid and paratyphoid (enteric) fever. Cochrane Database Syst Rev. 2017 May 26;5(5):CD008892. 10.1002/14651858.CD008892.pub2 28545155 PMC5458098

[4] Carey ME, Thi Nguyen TN, Tran DHN, Dyson ZA, Keane JA, Pham Thanh D, et al. The origins of haplotype 58 (H58) Salmonella enterica serovar Typhi. Commun Biol. 2024 Jun 28;7(1):775. 10.1038/s42003-024-06451-8 38942806 PMC11213900

[5] Arora P, Thorlund K, Brenner DR, Andrews JR. Comparative accuracy of typhoid diagnostic tools: A Bayesian latent-class network analysis. PLoS Negl Trop Dis. 2019 May 8;13(5):e0007303. 10.1371/journal.pntd.0007303 31067228 PMC6527309

[6] Lalremruata R, Chadha S, Bhalla P. Retrospective audit of the Widal test for diagnosis of typhoid Fever in pediatric patients in an endemic region. J Clin Diagn Res. 2014 May;8(5):DC22–5. 10.7860/JCDR/2014/7819.4373 24995178 PMC4079999

[7] Target product profile for laboratory tests for acute typhoid fever surveillance. Geneva: World Health Organization; 2025. Available from: https://www.who.int/publications/b/73092 [cited 2025 Oct 10].

[8] Vijay S, Gangakhedkar RR, Shekhar C, Walia K. Introducing a national essential diagnostics list in India. Bull World Health Organ. 2021 Mar 1;99(3):236–8. 10.2471/BLT.20.268037 33716346 PMC7941112

[9] Steingart KR, Ramsay A, Dowdy DW, Pai M. Serological tests for the diagnosis of active tuberculosis: relevance for India. Indian J Med Res. 2012 May;135(5):695–702. 22771604 PMC3401705

[10] Bal HB, Kar S, Otta S, Kumar S, Samal S, Pattnaik A, et al. Impact of cartridge-based nucleic acid amplification test (CBNAAT) introduction on the detection of pulmonary and extra-pulmonary tuberculosis in Bhubaneswar, Odisha. Indian J Pathol Microbiol. 2023 Oct-Dec;66(4):764–9. 10.4103/ijpm.ijpm_666_22 38084529

[11] Sapkota J, Roberts T, Basnyat B, Baker S, Hampton LM, Dittrich S. Diagnostics for typhoid fever: current perspectives and future outlooks for product development and access. Open Forum Infect Dis. 2023 Jun 2;10 Suppl 1:S17–20. 10.1093/ofid/ofad120 37274534 PMC10236505

[12] Andrews JR, Khanam F, Rahman N, Hossain M, Bogoch II, Vaidya K, et al. Plasma immunoglobulin A responses against 2 Salmonella Typhi antigens identify patients with typhoid fever. Clin Infect Dis. 2019 Mar 5;68(6):949–55. 10.1093/cid/ciy578 30020426 PMC6399438

[13] Fleming KA, Horton S, Wilson ML, Atun R, DeStigter K, Flanigan J, et al. The Lancet Commission on diagnostics: transforming access to diagnostics. Lancet. 2021 Nov 27;398(10315):1997–2050. 10.1016/S0140-6736(21)00673-5 34626542 PMC8494468

